# Structural Equation Modeling With Many Variables: A Systematic Review of Issues and Developments

**DOI:** 10.3389/fpsyg.2018.00580

**Published:** 2018-04-25

**Authors:** Lifang Deng, Miao Yang, Katerina M. Marcoulides

**Affiliations:** ^1^Department of Psychology, Beihang University, Beijing, China; ^2^Department of Psychology, University of Notre Dame, Notre Dame, IN, United States; ^3^School of Human Development and Organizational Studies in Education, University of Florida, Gainesville, FL, United States

**Keywords:** structural equation modeling, small sample size, parameter estimates, test statistics, stand errors

## Abstract

Survey data in social, behavioral, and health sciences often contain many variables (*p*). Structural equation modeling (SEM) is commonly used to analyze such data. With a sufficient number of participants (*N*), SEM enables researchers to easily set up and reliably test hypothetical relationships among theoretical constructs as well as those between the constructs and their observed indicators. However, SEM analyses with small *N* or large *p* have been shown to be problematic. This article reviews issues and solutions for SEM with small *N*, especially when *p* is large. The topics addressed include methods for parameter estimation, test statistics for overall model evaluation, and reliable standard errors for evaluating the significance of parameter estimates. Previous recommendations on required sample size *N* are also examined together with more recent developments. In particular, the requirement for *N* with conventional methods can be a lot more than expected, whereas new advances and developments can reduce the requirement for *N* substantially. The issues and developments for SEM with many variables described in this article not only let applied researchers be aware of the cutting edge methodology for SEM with big data as characterized by a large *p* but also highlight the challenges that methodologists need to face in further investigation.

## 1. Introduction

Many important attributes in the social, behavioral, and health sciences cannot be observed directly. Examples of such attributes include happiness, depression, anxiety, cognitive and social competence, etc. They are typically measured by multiple indicators that are often subject to measurement errors. Structural equation modeling (SEM) has become a major tool for examining and understanding relationships among latent attributes. Existing SEM methods are developed using asymptotics by assuming a large number of observations (*N*) and a small number of variables (*p*). However, with survey data or data collected using questionnaires, *p* can be rather large while *N* may be limited due to the high costs associated with obtaining a sufficient number of participants in the data collection. In such instances, blindly applying SEM methods developed using asymptotics can easily either result in misleading results or in unattainable parameter estimates due to non-convergences in computation. This article reviews the development of methods that aim to address small sample issues in SEM particularly when large numbers of variables are involved.

Note that a small or large sample size in SEM and other statistical modeling is relative to the number of variables, a method that works well with a small sample size is also expected to work well with a large number of variables. While our focus is mainly on methodology development, we will also discuss various recommendations on required sample size *N* in the SEM literature. These recommendations are typically based on limited simulation results using the method of normal-distribution-based maximum likelihood (NML). In particular, the requirement for *N* with the conventional NML method can be a lot more than expected to obtain reliable results, whereas new advances and developments can reduce the requirement for *N* substantially. We hope that our discussion of the various pros and cons of different methods will make researchers be aware of the potential problems and issues that might arise when using SEM with many variables, and the information offered would allow them to identify a method that is most suitable for their research when facing a data set with either a small *N* or a large *p*.

Let **S** be a sample covariance matrix based on a sample of size *N* with *p* variables. Currently, the most widely used method for SEM analysis is to fit **S** by a structural model using NML. Overall model evaluation is conducted by comparing the likelihood ratio statistic *T*_*ml*_ against the nominal chi-square distribution or by some fit indices such as root mean square error of approximation (RMSEA, Steiger and Lind, 1980), and/or the comparative fit index (CFI, Bentler, 1990). Standard errors of parameter estimates are obtained by inverting the normal-distribution-based information matrix. When data are normally distributed and the sample size is sufficiently large, the NML procedure is expected to perform well. In practice, data tend to be non-normally distributed (Micceri, 1989). Under such circumstance one may choose the Satorra-Bentler rescaled and adjusted statistics for overall model evaluation, and the sandwich-type covariance matrix for computing standard errors of parameter estimates (Satorra and Bentler, 1994). However, these procedures are not reliable when the number of variables *p* is relatively large, since their validity is justified by asymptotics. In particular, when *p* is large, the conventional SEM methods as implemented in most available software may fail to generate a set of parameter estimates due to the non-convergence that might occur during computation. Additionally, the likelihood ratio statistic may reject the correct model 100% of the time even when data are normally distributed.

The issue of small *N* with a relatively large *p* has been discussed by many authors in the extant literature (e.g., Barrett and Kline, 1981; Bentler and Chou, 1987; Jackson, 2001; de Winter et al., 2009; Xing and Yuan, 2017), in some cases aiming to reduce the requirement for *N* or to give a rule of thumb on the required sample size for properly conducting SEM. We try to provide an overview of up-to-date developments in methodology by addressing small sample issues in SEM. They include procedure for addressing the problems of near singular covariance matrix due to small *N*, obtaining more efficient parameter estimates with non-normally distributed data, improving the performance of test statistics, and procedures for more accurate standard errors (SEs). In the rest of the article, we first discuss various recommendations related to required sample sizes, including those proposed for exploratory factor analysis. Then we turn our attention to parameter estimation, where we discuss recent developments related to the use of ridge methods as well as penalized likelihoods. Overall model evaluation is discussed next, whereupon we review *ad-hoc* corrections as well as principled corrections to the likelihood ratio statistic and to the Satorra and Bentler's rescaled statistic. Standard errors are discussed subsequently, and includes a discussion of some advances that have been recently made. A real data example is presented next, contrasting conventional methods with recently developed new methods. Finally, we present some general recommendations, highlight some limitations, and conclude with emphasizing important remaining challenging issues in need of future attention.

## 2. *ad-hoc* recommendations concerning sample size

Many scholars have studied sample size issues in SEM and factor analysis. Earlier research noted that reasonable results could be obtained in SEM analyses when *N* is <200 (Gerbing and Anderson, 1985), or at least above 100 (Boomsma, 1985). While these recommendations were supported by Monte Carlo results, the number of variables in these studies was rather small. Bentler and Chou (1987) subsequently noted that sample size *N* should instead be considered relative to the number of parameters *q*, and the ratio of *N*:*q* can be as low as 5:1 for normally distributed data, and 10:1 for arbitrary distributions. However, recent studies have suggested that, as *p* increase, we in fact need an *N* that is much large than 200 in order for *T*_*ml*_ to perform as expected. In particular, it was found that at the nominal level of 5%, *T*_*ml*_ rejected a correct model 100% when *N* = 200 and *p* = 90 (Moshagen, 2012); and rejected correct models around 85% when *N* = 1, 000 and *p* = 120 (Shi et al., 2018). In addition, the studies by Jackson (2001), Moshagen and Shi et al. indicated that the behavior of *T*_*ml*_ is little affected by the number of parameters.

The issue of not having sufficiently large sample sizes has also been a major concern in exploratory factor analysis (EFA) since most well-known psychological scales contain many items, Within the context of EFA, the recommendations are quite varied, including for example for *N* to be above 100 and even up to above 1000 (Guilford, 1954; Kline, 1979; Gorsuch, 1983; Comrey and Lee, 1992), In terms of the ratio *N*/*p*, it has been suggested as needing to be anywhere from above 1.2 to up to 10 (Everitt, 1975; Cattell, 1978; Barrett and Kline, 1981; Gorsuch, 1983; Arrindell and van der Ende, 1985). MacCallum et al. (1999), however, argued that the necessary *N* is in fact dependent on other conditions in addition to *N*/*p*, including communality and the number of indicators per factor. These results were also confirmed by Preacher and MacCallum (2002) and Mundfrom et al. (2005). Interestingly, de Winter et al. (2009) noted that *N* can be much smaller than *p* if both the size of communality and the number of indicators per factor are high. However, the discussion and recommendations concerning the required sample size needed in EFA are mostly for the purpose of factor recovery^1^ instead of overall model evaluation or inference of parameter estimates.

While factor recovery in EFA might seem conceptually different from the issue of statistical inference in SEM or CFA, and the main focus in this article is model estimation and evaluation, our discussion and review will inevitably provide insight on inference issues in EFA as well. In particular, because factor recovery is closely related to standard errors of factor loading estimates, conditions or methods corresponding to more efficient parameter estimates will yield better factor recovery in EFA. For example, Yuan et al. (2010) have shown that standard errors (SEs) of factor loading estimates in CFA increase with the size of error variances, and decreases with the number of indicators per factor and the size of factor loadings other than that corresponding to the SE itself. We would expect these results also hold for standard errors of factor loading estimates in EFA. However, smaller values of SEs in EFA and more accurate estimates of SEs are two different concepts. Also, the conditions on *p* and *m* (the number of factors) that yield smaller SEs for parameter estimates are different from the conditions that lead to more reliable test statistics for overall model evaluation. For example, Shi et al. (2018) found that the distribution of *T*_*ml*_ is little affected by the number of indicators per factor (*p*/*m*), and the performance of *T*_*ml*_ becomes worse when *p* increases while holding *N* constant.

In summary, recommendations on required sample sizes in the literature of SEM and EFA are all simply *ad-hoc* conjectures. Although they might be based on small simulation studies with varied *N* but limited values of *p*, they are not justified statistically, nor generalizable to conditions with large *p*, as also noted by Yang et al. (2018).

## 3. Parameter estimation

A SEM model can be formulated according to theory or information gained at the exploratory stage with EFA. Once a model is formulated, we need to obtain parameter estimates before model evaluation. We discuss several major methods in this section, including normal distribution based maximum likelihood (NML), generalized least squares (GLS), the Bayesian approach, penalized likelihood, and some related methods to yield more stable/efficient parameter estimates.

### 3.1. NML and ridge ML

The most widely used method for parameter estimation in SEM is NML, which is equivalent to minimizing the discrepancy function

(1) $$\begin{array}{l} F_{ml}\left(\theta \right)=\text{tr}\left(\text{S}\Sigma^{-1}\left(\theta \right)\right)-\log|\text{S}\Sigma^{-1}\left(\theta \right)|-p \end{array}$$

for estimating **θ**, where **Σ**(**θ**) is the structural model. Unlike in linear regression where there is an analytical formula for estimating the regression coefficients, we have to use an iterative procedure to minimize *F*_*ml*_(**θ**), and the Fisher-scoring algorithm (see Lee and Jennrich, 1979) is typically used for such a purpose. Small *N* or large *p* can cause various problems when minimizing *F*_*ml*_(**θ**), including near singular covariance matrices due to not having enough distinct observations and slower convergence due to large sampling errors. When a sample does not contain a sufficient number of distinct cases, the sample covariance matrix **S** is near singular (not full rank). Then, the iteration process for computing the NML estimates can be very unstable, and it may take literally hundreds of iterations to reach a convergence (Yuan and Bentler, 2017). When **S** is literally singular, equation (1) is not defined, and other methods for parameter estimation will likely break down as well. Even when the sample size is quite large, **S** can be near singular in real data analysis due to multi-collinearity (Wothke, 1993), especially when *p* is large. A great deal of research has been directed to address the problems encountered with near singular covariance matrices, and to increase the chance and speed of convergence in computation at the same time.

When **S** is singular, the program LISREL (Jöreskog and Sörbom, 1993) provides an option of ridge SEM by replacing the **S** in the NML discrepancy function in Equation (1), with **S** + *k*diag(*s*_11_, …, *s*_*pp*_), where *k* > 0 and *s*_*jj*_ is the sample variance of the *j*th variable. However, statistical properties of the resulting ridge parameter estimates and test statistics have not been obtained analytically or asymptotically. Through empirical studies, McQuitty (1997, p. 251) concluded that “there appears to be ample evidence that structural equation models should not be estimated with LISREL's ridge option unless the estimation of unstandardized factor loadings is the only goal.” A ridge technique that is different from the one implemented in LISREL was developed by Yuan and Chan (2008). They proposed to replace **S** in equation (1) by **S**_*a*_ = **S** + *a***I** and recommended choosing *a* = *p*/*N*. Let the resulting discrepancy function be denoted by *F*_*mla*_(**θ**), and call the procedure of minimizing *F*_*mla*_(**θ**) ridge ML. Yuan and Chan (2008) showed that both the speed of convergence and convergence rate of the Fisher-scoring algorithm in ridge ML are much higher than in ML. They also showed that, with smaller *N*s, ridge ML yields consistent and more efficient parameter estimates ${\hat{\theta }}_{a}$ than ML even when data are normally distributed. They further proposed a rescaled statistic and sandwich-type SEs for model and parameter evaluation. However, with this approach, the inferences are still based on asymptotics. Furthermore, with typically non-normally distributed data (Micceri, 1989), minimizing *F*_*mla*_(**θ**) may not generate most efficient parameter estimates of **θ**, because the function does not account for possible skewness and kurtosis in the sample.

In item factor analysis with ordinal data, the polychoric correlation matrix **R** tends to be nonpositive definite because different elements are computed using different pairs of variables. One method of item factor analysis is to treat **R** as a covariance matrix in the NML based method, and constrain all the diagonal elements of the structural model implied covariance matrix **Σ** at 1.0. This method has been recommended by Lei (2009) for SEM analysis when the sample size *N* is not large enough. But it fails when **R** is not positive definite. Yuan et al. (2011) generalized the ridge ML method in Yuan and Chan (2008) for continuous data to ordinal data by fitting a structural model to **R**_*a*_ = **R** + *a***I** via minimizing NML discrepancy function *F*_*mla*_. Empirical results indicated that ridge ML for ordinal data yields much more efficient parameter estimates than ML for ordinal data when modeling polychoric correlations. While Yuan et al. (2017) did not compare ridge ML directly with the widely used methods of least squares (LS) and diagonally weighted least squares (DWLS), we would expect ridge ML to yield more efficient parameter estimates than LS and DWLS, especially when *N* is small and *p* is relatively large. However, like for NML with continuous data, the sampling property of **R** is not accounted for in the stage of parameter estimation with the ridge ML method.

### 3.2. GLS and ridge GLS

Arruda and Bentler (2017) studied the normal-distributed-based generalized-least-squares (GLS) method, and proposed to replace the sample covariance matrix in the GLS weight matrix by a regularized covariance matrix. While their development is to improve the conditions of the weight matrix, they mostly focused on the performance of GLS-type test statistics for overall model evaluation with normally distributed data. We would expect that this regularized GLS method would yield more efficient parameter estimates than NML or GLS with large *p*. Further study is needed.

A well-known development that considers the sampling variability of the sample covariance matrix **S** is the generalized-least-squares (GLS) method developed by Browne (1984), which is also called the asymptotically distribution free (ADF) method. Let **s** be the vector of non-duplicated elements in **S**, **σ**(**θ**) be the structured counterpart of **s**, and $\hat{\Gamma }$ be a consistent estimator of the covariance matrix of *N*^1/2^**s**. The GLS/ADF discrepancy function is given by

(2) $$\begin{array}{l} F_{gls}\left(\theta \right)={\left(\text{s}-\sigma \left(\theta \right)\right)}^{\prime }\text{W}\left(\text{s}-\sigma \left(\theta \right)\right), \end{array}$$

where $\text{W}={\hat{\Gamma }}^{-1}$, with $\hat{\Gamma }$ being the sample 4th-order moment matrix (Mooijaart and Bentler, 2011). The GLS/ADF parameter estimates are obtained by minimizing the function *F*_*gls*_(**θ**) in equation (2). While the GLS/ADF method enjoys the property of yielding asymptotically most efficient estimator among all methods of modeling **S**, its performance is rather poor unless the sample size *N* is rather large and *p* is relatively small. Further studies show that this is because $\hat{\Gamma }$ is very unstable, especially when $\hat{\Gamma }$ is near singular with a large *p* and a relatively small *N* (Yuan and Bentler, 1998; Huang and Bentler, 2015).

Note that the function *F*_*gls*_(**θ**) in equation (2) becomes the LS discrepancy function when **W** is replaced by the identity matrix **I**. In contrast to the instability of $\hat{\Gamma }$, the weight matrix **I** in the LS method is most stable because it does not depend on data. However, there is no mechanism in the LS method to account for the variances of the elements in **S**, and consequently the LS estimator does not possess the desired asymptotic properties of the GLS/ADF estimator. Considering the pros and cons of LS and ADF method, Yuan and Chan (2016) proposed a ridge GLS method in which the weight matrix **W** in Equation (2) is replaced by ${\text{W}}_{a}={\left[a\hat{\Gamma }+\left(1-a\right)\text{I}\right]}^{-1}$, where *a* is a scalar that can be tuned according to certain conditions. Clearly, GLS/ADF corresponds to *a* = 1 while LS corresponds to *a* = 0. We can choose an *a* in between to yield the most efficient parameter estimates. Results in Yuan and Chan (2016) indicate that, with typically non-normally distributed data in practice, ridge GLS yields uniformly more efficient parameter estimates than LS, GLS/ADF, and NML. While ridge GLS enjoys various advantages over other well-known methods, it involves the determination of the tuning parameter *a*. Currently, there does not exist an effective method for choosing *a* to yield most efficient parameter estimates. Also, as we will discuss in the next section, the test statistics following ridge methods also need to be calibrated, especially with large *p* and small *N*.

### 3.3. The Bayesian method

In addition to ML and GLS, another major method for parameter estimation is the Bayesian approach. In particular, for some nonlinear models (e.g., models involving interactions among latent variables), the likelihood function of the model parameters might be hard to specify or becomes too complicated to work with. It is relatively easy to specify a conditional distribution of the parameters via data augmentation (Gelman et al., 2014). Thus, one might be able to obtain results close to those by ML or GLS using the Bayesian approach that is facilitated by Gibbs sampling or Markov chain Monte Carlo (MCMC). Another advantage of the Bayesian approach is that one can include prior information by properly specifying prior distributions for the model parameters. However, properly specifying prior distributions needs skills, especially when the prior information does not come in the form of an inverted Wishart distribution or inverted gamma distribution that are needed in most developments of Bayesian methodology (Scheines et al., 1999; Lee, 2007), and inaccurate specification of the prior distributions can result in biased estimates (Baldwin and Fellingham, 2013; Depaoli, 2014; McNeish D., 2016). While the covariance matrix **S**_*a*_ = **S** + *a***I** in ridge ML can also be regarded as a Bayesian estimate by specifying a prior distribution for the saturated covariance matrix, the effect of *a* is removed from the estimates of error variances (Yuan and Chan, 2008). This is why ridge ML yields more accurate parameter estimates than NML even when data are normally distributed.

Unlike the methods of ML and GLS that are justified by asymptotics, the modern Bayesian approach to estimation and inference is based on sampling from the posterior distribution. Thus, the Bayesian method has been suggested to deal with issues for for small *N*. Indeed, the MCMC method has been shown to outperform ML and GLS in small sample contexts (Lee and Song, 2004; Zhang et al., 2007; Moore et al., 2015; van de Schoot et al., 2015; McNeish D. M., 2016). However, informative priors are used in these studies. In particular, when *N* is small, the priors are expected to dominate the results. Actually, one may get satisfactory results even when *N* = 0 if accurate priors are specified. Thus, to a certain degree, the small sample advantage of a Bayesian method is subjective. With flat or Jeffreys noninformative priors, Bayesian methods are expected to yield equivalent results to ML. However, empirical results do not endorse the Bayesian methods with small *N* (Baldwin and Fellingham, 2013; McNeish D. M., 2016).

In conclusion, when prior information is indeed available and one can include it in the current study via an accurate specification of priors and the distribution of current data given the parameter, Bayesian method is preferred and will be able to successfully address the problem for SEM with small *N* and/or large *p*. One needs to be cautions when either of the specifications is not proper. In particular, Bayesian methods “will not address the small sample issue and that ML with small sample alterations typically produce estimates with quality that can equal or often surpass MCMC methods that do not carefully consider the prior distributions” (McNeish D. M., 2016, p. 753).

### 3.4. Penalized likelihood

The issue of large *p* and small *N* also poses challenges to other conventional statistical methods. In the literature of regression analysis, lasso methodology has been shown to be a viable method with big data or when *p* is rather large but *N* is not sufficiently large (Tibshirani, 1996). As a generalization of ridge regression, the idea of lasso is to squeeze parameters with small values so that they are equivalent to being removed from the model. When there are too many predictors to consider and when their relevance is unclear, lasso regression provides a viable tool for conducting regression analysis and variable selection at the same time. Lasso methodology has also been generalized to SEM via penalized likelihood (Jacobucci et al., 2016; Huang et al., 2017). However, although there can be many items with survey data, SEM is generally conceptualizered as a confirmatory methodology. In particular, it is expected that both the measurement and the structural parts of an SEM model be established in priori, and while the model parameters are freely estimated they are not free to be removed, simply because the resulting model might correspond to a completely different theoretical hypothesis. Also, the size of a parameter in SEM is scale dependent. The estimates with smallest values might be statistically most significant.

Parallel to factor rotation, lasso methodology might be a more useful technique for exploratory factor analysis when applied to standardized variables, because the scales of the measured variables become irrelevant. While the corresponding parameter estimates with standardized variables are more comparable, it does not imply that their standard errors also are comparable or become irrelevant (Cudeck, 1989). One still needs to be cautious when using the lasso methodology for big data in SEM or factor analysis, especially when items that are theoretically important for measuring an underlying construct might have smaller loadings.

In summary, various methods have been proposed in SEM to yield more accurate/efficient parameter estimates. Currently, the ridge ML and ridge GLS appear to be the most promising methods. In particular, when combining ridge ML with robust transformation (Yuan et al., 2000), ridge ML may in fact yield estimates that are close to full information maximum likelihood estimates. Further study in this direction is clearly needed.

## 4. Test statistics

In addition to making parameter estimation difficult, a large *p* also causes problems to the overall model evaluation, which is considered by many researchers a key aspect of SEM (e.g., Marcoulides and Yuan, 2017). Model evaluation has also gained more extensive studies than other aspects of SEM, simply because any elaboration on parameter estimates or causal relationship among the variables is conditional upon determination of an adequate model. Because there are many extensive developments in this direction that have appeared in the literature, here we only discuss the pros and cons of methods connected to the issue of the effect of small *N* and/or large *p*. Most of these studies on advancing model inference in SEM within this context follow two directions. One is to account for non-normally distributed data; and the other is to account for small sample sizes or large number of variables. Of course, because statistics that account for non-normally distributed data also face the challenge of large number of variables, we inevitably also focus our review on their behaviors with small *N* and/or large *p*.

### 4.1. Correction to *T*_*ml*_ under the normality assumption

The most widely used test statistic in SEM is *T*_*ml*_ = (*N*−1)*F*_*ml*_, mostly because it is the default statistic in available software, not because normally distributed data are common or *T*_*ml*_ provides more reliable model evaluation. Under the normality assumption and a correct model, *T*_*ml*_ approaches the nominal chi-square distribution $\chi_{df}^{2}$ as the sample size *N* increases while *p* is fixed. However, this result does not tell us how large *N* needs to be at a given value of *p* for *T*_*ml*_ to approximately follow $\chi_{df}^{2}$. As we noted earlier, the statistic *T*_*ml*_ can reject the correct model 100% at the nominal level of 5% even when data are normally distributed. While there are a lot of efforts to improve the performance of *T*_*ml*_ by many authors, most are ad hoc corrections rather than principled ones. Consequently, the behavior of the corrected statistics can vary as conditions change.

It is a general and well-known phenomenon that the likelihood ratio statistic tends to reject the correct model more often than expected when *N* is not sufficiently large, not just in SEM. As a consequence, statisticians have developed a systematic approach for correcting the likelihood ratio statistic, and it is called the Bartlett correction (Bartlett, 1937, 1954; Box, 1949; Lawley, 1956). Wakaki et al. (1990) obtained the Bartlett correction to *T*_*ml*_ for a class of covariance structural models with normally distributed data. However, the corrected statistic is rather complicated even for a relatively simple model, and it is impractical to implement the Bartlett correction on *T*_*ml*_ for general SEM models. In the context of exploratory factor analysis (EFA), Bartlett (1951, see also Lawley and Maxwell, 1971, p. 36) proposed a simplified formula to correct the likelihood ratio statistic, which is to replace (*N*−1) in *T*_*ml*_ with *N*_*b*_ = *N*−(2*p* + 11)/6 − 2*m*/3, where *m* is the number of factors. Because the corrected statistic, *T*_*mlb*_ = *N*_*b*_*F*_*ml*_, is easy to implement, Nevitt and Hancock (2004, see also Fouladi, 2000) proposed to apply the simple correction to confirmatory factor analysis (CFA) and SEM, where *m* is still the number of latent factors. However, studies by Nevitt and Hancock (2004) and Herzog et al. (2007) indicate that type I errors with $T_{mlb}\sim \chi_{df}^{2}$ in SEM tend to be much lower than the nominal level.

Considering that the number of free parameters in SEM is much smaller than that in EFA when *m* is large, Yuan (2005) proposed to replace (*N* − 1) in the definition of *T*_*ml*_ with *N*_*y*_ = *N*−(2*p*+13)/6−*m*/3. However, this proposal is only a heuristic rather than one that is statistically justified. A more complicated correction was originally offered by Swain (1975), who proposed to replace (*N* − 1) in *T*_*ml*_ by

$$\begin{array}{l} N_{s}=N-1-\left[p\left(2p^{2}+3p-1\right)-h_{q}\left(2h_{q}^{2}+3h_{q}-1\right)\right]/\left(12df\right), \end{array}$$

where $h_{q}=\left[{\left(1+8q\right)}^{1/2}-1\right]/2$ and *q* is the number of free parameters in the structural model. Studies by Fouladi (2000), Herzog et al. (2007) and Herzog and Boomsma (2009) indicate that the performance of test from best to worst are *T*_*mls*_ = *N*_*s*_*F*_*ml*_, *T*_*mly*_ = *N*_*y*_*F*_*ml*_, and *T*_*mlb*_. Although the performance of *T*_*mls*_ is potentially promising, the correction is not statistically justified.

Parallel to the Bartlett correction, Yuan et al. (2015) developed a procedure that involved an empirical correction. In particular, they proposed to estimate the coefficient **β** in

$$\begin{array}{l} T_{mle}=\left(N-{\text{c}}^{\prime }\beta \right)F_{ml} \end{array}$$

by matching the empirical mean of *T*_*mle*_ with the nominal degrees of freedom or the mean of $\chi_{df}^{2}$, where **c** is a vector whose elements are different combinations of *p*, *q*, and *m*. Using Monte Carlo results across 342 conditions of *N*, *p*, *q*, and *m*, they estimated the vector **β** by maximum likelihood. One of the statistics they recommended is *T*_*mle*_ = *N*_*e*_*F*_*ml*_, where *N*_*e*_ = *N* − (2.381 + 0.361*p* + 0.003*q*). Yuan et al. (2015) noted that *T*_*mle*_ can be properly used when *N* > max(50, 2*p*). Recently, Shi et al. (2018) conducted a rather comprehensive simulation study and showed that *T*_*mle*_ performed better than *T*_*ml*_, *T*_*mlb*_, and *T*_*mls*_. However, type I error rates of *T*_*mle*_ can still be inflated when *p* is extremely large (e.g., *p* = 90), even when *N* = 200. They noted that for normally distributed data *N* needs to be >4*p* in order for *T*_*mle*_ to properly control type I errors if *p* is over 100.

### 4.2. Corrections to test statistics that account for non-normality

In addition to correcting *T*_*ml*_ for normally distributed data with small *N* and/or large *p*, various developments on test statistics with non-normally distributed data were also made, including modifying the statistic *T*_*ml*_ following NML as well as working with GLS, ridge GLS or a robust estimation method. We discuss next their properties with small *N* as well as those of their modified versions.

The most widely used statistics that account for non-normality are the rescaled statistic *T*_*rml*_ and the adjusted statistic *T*_*aml*_, developed by Satorra and Bentler (1994). The statistic *T*_*rml*_ has the property that its mean asymptotically equals that of the nominal chi-square distribution $\chi_{df}^{2}$, and the statistic *T*_*aml*_ has the property that both its mean and variance asymptotically equal those of the approximating chi-square distribution $\chi_{df_{a}}^{2}$. Note that the value of *df*_*a*_ (the degrees of freedom) for the reference distribution of *T*_*aml*_ is determined by both the model and the underlying population distribution, and needs to be estimated in practice. Among the two statistics, *T*_*rml*_ is more widely used and is called a robust chi-square statistic by some authors (Bentler and Yuan, 1999). Although the exact distribution of neither *T*_*rml*_ nor *T*_*aml*_ is known even asymptotically, Monte Carlo results in Fouladi (2000) at *p* = 6 and 12, and in Hu et al. (1992) at *p* = 15 indicate that they perform reasonably well for medium to large *N*. However, there exists evidence that *T*_*rml*_ and *T*_*aml*_ do not work well with small *N* (Bentler and Yuan, 1999; Nevitt and Hancock, 2004). In particular, when *p* is relatively large, results in Yuan et al. (2017) suggested that *T*_*rml*_ can reject the correct model from 0 to 100% while the nominal rate is 5%.

Earlier studies indicated that *T*_*rml*_ tend to over-reject the correct model when *N* is not sufficiently large (e.g., Hu et al., 1992; Bentler and Yuan, 1999). Many *ad-hoc* corrections have been proposed to correct such behavior, including $T_{rml}^{\left(b\right)}$ obtained by replacing the (*N*−1) in the formulation of *T*_*rml*_ with *N*_*b*_, which is the formula proposed by Bartlett (1951) for correcting the behavior of *T*_*ml*_ in EFA. Aiming to improve the behavior of *T*_*rml*_ in over-rejecting corrected models at smaller *N*, Jiang and Yuan (2017) proposed four statistics to further modify the behavior of *T*_*rml*_. However, these statistics may reject the correct model 0 times in some conditions. Yang et al. (2018) studied 10 modifications of *T*_*rml*_, including $T_{rml}^{\left(b\right)}$, and the four proposed in Jiang and Yuan (2017). Using the average of the absolute deviations from the nominal level as a criterion, results in Yang et al. (2018) indicate that $T_{rml}^{\left(b\right)}$ performed the best across 604 conditions of *N*, *p*, and different population distributions. But still $T_{rml}^{\left(b\right)}$ rejected correct models from 0 to 96%. In particular, when *N* is small and *p* is relatively large, the rejection rate by $T_{rml}^{\left(b\right)}$ for correct models is close to 100% with normally distributed data, and 0% when data follow a population distribution with heavy tails. Thus, $T_{rml}^{\left(b\right)}$ is not a reliable test statistic for SEM when *p* is large and/or *N* is relatively small.

Comparing to *T*_*rml*_, fewer studies for the adjusted statistic *T*_*aml*_ indicate that it tends to under-reject correct models, and perform rather well when *p* is relatively small (Fouladi, 2000). However, there is a noticeable lack of studies focusing on *T*_*aml*_ with respect to type I error control at relatively large *p*. Nevitt and Hancock (2004) noted that the resulting statistic of replacing the (*N* − 1) in the formulation of *T*_*aml*_ by *N*_*b*_ (Bartlett's formula for EFA) does not work well.

Note that *T*_*rml*_ and *T*_*aml*_ of Satorra and Bentler are derived from the principles of mean and mean-and-variance correction (e.g., Welch, 1938; Rao and Scott, 1984), and they are expected to work well in practice, especially when *p* or the degrees of freedom are large (e.g., Yuan and Bentler, 2010). Yang et al. (2018) recently examined the causes for *T*_*rml*_ and *T*_*aml*_ to fail to control type I errors, and found that neither *T*_*aml*_ nor *T*_*rml*_ possess the properties to which they are entitled asymptotically. That is, their means and variances can be far from those of their reference distributions. Even for normally distributed data, the mean of *T*_*rml*_ can be hundreds of times greater than that of the nominal chi-square distribution in standardized units when *p* is large but *N* is not sufficiently large. For non-normally distributed data, the mean of *T*_*rml*_ can also be much smaller than that of the nominal chi-square distribution when both *p* and *N* are large. Also, there are many conditions under which the mean of *T*_*aml*_ is much greater than that of its reference chi-square distribution $\chi_{df_{a}}^{2}$ whereas the standard deviation of *T*_*aml*_ is much smaller than that of $\chi_{df_{a}}^{2}$. This is because the mean and mean-and-variance corrections for obtaining *T*_*rml*_ and *T*_*aml*_ are implemented via standard asymptotics, which fail when *p* is relatively large. Yang et al. (2018) noted that mean and mean-and-variance corrections are still expected to work well with big data but we have to use alternative methods instead of those based on asymptotics to implement them.

A recent development in correcting the behavior of *T*_*rml*_ is given by Yuan et al. (2017). Parallel to obtaining *T*_*mle*_, they replaced the term (*N*−1) in the formulation of *T*_*rml*_ with a scalar $N_{c}=N-{\text{c}}^{\prime }\beta$. However, the elements of the vector **c** contain covariates that reflect the underlying population distribution of the sample in addition to various nonlinear functions of *N*, *p*, *q*, and *df*. The coefficients in **β** are estimated so that the corrected statistic $T_{rml}^{\left(c\right)}=N_{c}F_{rml}$ has a mean approximately equal to that of the nominal chi-square distribution, not according to asymptotics but according to empirical results. With many conditions of *N*, *p*, *m*, *q*, and population distribution, they evaluated different formulations of *N*_*c*_, and recommended a statistic containing 20 predictors, denoted it as $T_{rml}^{\left(c20\right)}$. They further conducted an independent simulation study to evaluate $T_{rml}^{\left(c20\right)}$, and found that it performed substantially better than $T_{rml}^{\left(b\right)}$. In particular, for normally distributed data with *p* ranging from 20 to 80, the rejection rates of $T_{rml}^{\left(c20\right)}$ range from 2.4 to 7.6%, the rejection rates of $T_{rml}^{\left(b\right)}$ range from 2.2 to 57.8%, and those of *T*_*rml*_ range from 4.8 to 100%. For data that follow elliptical distributions, the rejection rates of $T_{rml}^{\left(c20\right)}$ range from 5.8 to 14%, those of $T_{rml}^{\left(b\right)}$ range from 0 to 5.4%, and those of *T*_*rml*_ range from 0 to 95.4%. We may think that $T_{rml}^{\left(b\right)}$ performed well for the condition of elliptical population distributions, however, its rejection is 0% for many of the conditions studied, and type I error rates do not tell how bad its performance is for these conditions once the rates are equal to zero.

The statistics we discussed so far, as listed in the first part of Table 1, are all derived from the normal-distribution-based maximum likelihood (NML), and the parameter estimates under these statistics are the same. In particular, unless data are normally distributed, NML does not account for the underlying population distribution in estimating model parameters. As we noted in the previous section, the GLS/ADF method uses the inverse of a consistent estimator of the covariance matrix of *N*^1/2^**s** as the weight matrix, which directly accounts for the underlying population distribution. In addition to yielding asymptotically most efficient parameter estimates among all the methods of modeling **S**, the corresponding statistic *T*_*gls*_ = (*N*−1)*F*_*gls*_ also asymptotically follows the nominal chi-square distribution. With *p* = 15 manifest variables, results in Hu et al. (1992) suggested that *T*_*gls*_ performs as expected at *N* = 5, 000, and it rejects the correct model 100% at *N* = 150. Thus, as *p* increases, the requirement for sample size by $T_{gls}\sim \chi_{df}^{2}$ to perform reasonably well is even more demanding than $T_{rml}\sim \chi_{df}^{2}$ or $T_{aml}\sim \chi_{df}^{2}$.

**Table 1:** Test statistics and their applicability.

**Statistic**	**Source**	**Applicability**		
		***p***	***N***	**Distribution**
*T*_*ml*_	Likelihood ratio	S	L	NM
*T*_*mlb*_	Nevitt and Hancock, 2004	S	L	NM
*T*_*mly*_	Yuan, 2005	S	L	NM
*T*_*mls*_	Swain, 1975	S to M	M to L	NM
*T*_*mle*_	Yuan et al., 2015	S to M	M to L	NM
*T*_*rml*_	Satorra and Bentler, 1994	S	L	NM & NNM
*T*_*aml*_	Satorra and Bentler, 1994	S	L	NM & NNM
$T_{rml}^{\left(b\right)}$	Nevitt and Hancock, 2004	S	L	NM
$T_{rml}^{\left(c20\right)}$	Yuan et al., 2017	S to M	M to L	NM & NNM
*T*_*gls*_	Browne, 1984	S	vL	NM & NNM
*T*_*cgls*_	Yuan and Bentler, 1997a	S	M to L	NM & NNM
*T*_*F*_	Yuan and Bentler, 1999	S	M to L	NM & NNM

S, small; M, medium; L, large; vL, very large; NM, normal; NNM, nonnormal

*The two underlined statistics are recommended*.

Statistics that are less demanding for sample size following the GLS/ADF estimation have also been developed over the years. One is a corrected GLS/ADF statistic *T*_*cgls*_ = *T*_*gls*_/(1+*F*_*gls*_), which was obtained by Yuan and Bentler (1997a) via estimating the covariance matrix of *N*^1/2^**s** using the cross-product of residuals instead of using 4th-order sample moments. Another is an *F*-statistic (Yuan and Bentler, 1999)

(3) $$\begin{array}{l} T_{F}=\frac{\left(N-df\right)F_{gls}}{df}, \end{array}$$

which is referred to an *F*-distribution with *df* and *N* − *df* degrees of freedom. In addition, Yuan and Bentler (1998) also proposed statistics that are based on residuals $\text{s}-\hat{\sigma }$, where the parameter estimates $\hat{\theta }$ in $\hat{\sigma }=\sigma \left(\hat{\theta }\right)$ might be obtained by NML or least squares. In addition to being asymptotically distribution-free, these statistics perform reasonably well for medium to large sample sizes (Fouladi, 2000; Nevitt and Hancock, 2004). However, when *N* is relatively small, *T*_*F*_ tends to over-reject correct models and *T*_*cgls*_ tends to under-reject correct models (Bentler and Yuan, 1999). Also, we need to have *N* > *df* for *T*_*F*_ and *T*_*cgls*_ to be properly defined, and a much larger *N* is needed for them to closely follow their nominal distributions. Because the value of *df* tends to increase fast with *p*, the statistics *T*_*F*_ and *T*_*cgls*_ are not solutions to inference issues for SEM when *p* is large.

### 4.3. Other GLS-type test statistics

As described in the previous section, Arruda and Bentler (2017) studied a regularized normal-distribution-based GLS method and found that one of the resulting test statistics performed rather well at *p* = 15 and *N* = 60 for normally distributed data. While this statistic is expected to work better than its unregularized version as well as *T*_*ml*_ with larger *p*, it is clear that further study on its performance with large *p* and small *N* as well as non-normally-distributed data is needed.

Following the NML estimation method, a GLS-type statistic is obtained when the weight matrix is obtained by the estimated covariance matrix instead of the sample covariance matrix. This statistic is called “the normal theory RLS chi-square" statistic in EQS (Bentler, 2006). A ridge version of this statistic obtained by fitting the polychoric correlation matrix with ordinal data was studied in Yuan et al. (2011). They found that the rescaled and adjusted versions of the ridge RLS type statistics performed much better than the rescaled and adjusted versions of the ridge ML type statistics. Because the formulation of the ridge RLS type statistic is very close to the regularized GLS statistic studied in Arruda and Bentler (2017), in general we would expect that the rescaled and adjusted ridge RLS type test statistics to perform better than the rescaled and adjusted ridge ML statistics with large *p*. Of course, additional research is clearly needed in this direction before any definitive conclusion can be drawn. As pointed by Huang and Bentler (2015) and noted in the previous section, the poor performance of the GLS/ADF statistic *T*_*gls*_ is closely related to the condition of the weight matrix $\text{W}={\hat{\Gamma }}^{-1}$ in Equation (2), especially if $\hat{\Gamma }$ is near singular when *N* is small relative to *p*. Using the idea of principal components, Chun et al. (2017) proposed to test the structural model by ignoring the directions corresponding to the smallest eigenvalues of $\hat{\Gamma }$. The resulting statistic is shown to control type I errors better. But the null hypothesis of the test is different from that of other test statistics reviewed in so far, because the test is unable to identify misspecifications in the direction represented by the vectors corresponding to the smallest eigenvalues of $\hat{\Gamma }$.

In summary, much research has focused on trying to obtain more reliable test statistics for overall model evaluation in cases with large *p* and/or relatively small *N*. Most of the obtained statistics are justified by either asymptotics or simple *ad-hoc* corrections to *T*_*ml*_ or *T*_*rml*_. Currently, the most reliable statistic for normally distributed data with many variables is the statistic *T*_*mle*_, developed in Yuan et al. (2015). The most reliable statistic for data with many variables that are possibly non-normally distributed is the statistic $T_{rml}^{\left(c20\right)}$, developed in Yuan et al. (2017). These two statistics are underlined in Table 1.

While ridge ML, ridge GLS and robust methods have been shown to yield more efficient parameter estimates, there is little development aimed at improving the performance of the rescaled and adjusted test statistics following these methods (Yuan and Chan, 2008, 2016; Tong et al., 2014).

## 5. Standard errors of parameter estimates

Standard errors (SEs) for parameter estimates are also key elements in SEM although they are secondary compared to parameter estimates or test statistics for overall model fit evaluation. In particular, once a model is deemed adequate following a proper estimation method, the meaning of the estimated values of the parameters must then be properly elaborated and explained. It is in this context that, accurate SE estimates are essential for proper interpretations. However, compared to test statistics or parameter estimation, much less research has focused on ways to improve the estimation of SEs. While a formula for computing SEs is typically provided with each method of parameter estimation in SEM, the formula is mainly justified by asymptotics, and may not work well when the number of variables is large. We review existing approaches to estimating SEs in this section, and point out their potential problems with small *N* and/or large *p*.

Coupled with the test statistic *T*_*ml*_, standard errors following the NML method in SEM are computed by inverting the corresponding information matrix, as is given in the default output of most SEM software. Such obtained SEs are consistent when the normality assumption literally holds and the model is correctly specified. When either the normality assumption is violated or when the model is misspecified, SEs based on the information matrix are not consistent (Yuan and Hayashi, 2006). We are not aware of any study to date on their accuracy for large *p* or small *N*. While there is a general interest in the performance of the SEs based on the information matrix, because data commonly collected in social and behavioral science are typically non-normally distributed (Micceri, 1989), additional effort on improving the information-matrix-based SEs may not ultimately be worth the investment.

The NML method is widely used in practice regardless of the distribution of the data. This is because there are not many multivariate distributions to choose from, and we typically do not know the population distributions. Following NML estimation, SEs based on the so-called sandwich-type covariance matrices have been proposed to account for violations of normality (White, 1981; Bentler, 1983; Shapiro, 1983; Browne, 1984), and they have been implemented in most SEM software. Such SEs are also called robust SEs in the SEM literature, parallel to the rescaled statistic *T*_*rml*_. However, the sandwich-type SEs as implemented in statistical packages are based on the assumption of a correctly specified model, because the formula becomes rather complicated otherwise (Yuan and Hayashi, 2006). While it is unlikely that a researcher can specify a model that is literally correct in practice, sandwich-type SEs are close to being consistent if the model is deemed adequate. However, consistency does not tell us how good the SE estimates are in a given application. While there are limited studies on the performance of sandwich-type SEs in SEM (Yuan and Bentler, 1997b; Yuan and Chan, 2016), there is a great deal of evidence that sandwich-type SEs are not reliable when *p* is large and *N* is not sufficiently large in other contexts (MacKinnon and White, 1985; Long and Ervin, 2000; Yang and Yuan, 2016). In particular, for regression models with heteroscedastic variances, various corrections to SEs have been proposed (see e.g., Cribari-Neto, 2004), however, because these may not be directly generalizable to the SEM context, it is evident that further research is needed on this topic.

In addition to yielding a statistic *T*_*gls*_ that asymptotically follows the nominal chi-square distribution, the GLS/ADF method also generates a formula that yields consistent SEs for the GLS estimates. However, like *T*_*gls*_, it needs a rather large sample size for the formula-based SEs to match those of the empirical ones. When *p* is large while *N* is not sufficiently large, the SEs computed by GLS/ADF formula are too small. Yuan and Bentler (1997b) proposed a correction to the formula of the covariance matrix of the GLS/ADF estimator. While the corresponding corrected SEs are much improved over the uncorrected ones, but they are still under-estimated, especially when *N* is small. Further improvement over the corrected SEs is possible. But the GLS/ADF estimator can be rather inefficient for small *N*, and the additional effort needed to improve the estimates of SEs for not efficient parameter estimates may not be worthwhile.

The bootstrap method has also been shown to yield reliable SEs, and is especially valuable when formula-based SEs are not available (Efron and Tibshirani, 1993). In particular, a model does not need to be literally correct in order for the bootstrap method to yield consistent SEs (Yuan and Hayashi, 2006). Indeed, since the bootstrap methodology is based on resampling that accounts for both the sample size and empirical distribution, we would expect it to work reliably regardless of the values of *N* and *p*. Currently, however, we are not aware of any study that verifies the validity of the bootstrap methodology for SEM with large *p* and small *N*.

In summary, few studies have focused on improving estimates of standard errors in SEM with large *p*. Although the bootstrap methodology appears promising, it is not a substitute for analytical formulas. This is because the bootstrap methodology is essentially Monte Carlo simulation with empirical data. It takes time to estimate the parameters in conducting the simulation with SEM models, especially when *p* is large. The issue of non-convergence with parameter estimation discussed earlier can be a serious problem for the bootstrap methodology since there exist systematic differences between converged and non-converged replications (Yuan and Hayashi, 2003), and SEs based on only the converged replications might under-estimate the true SEs. Since efficient parameter estimates are fundamental to statistical inference, future research should focus on developing more reliable SEs perhaps by focusing on the development of methods (such as ridge GLS and robust methods) that yield more efficient parameter estimates.

## 6. A real data example

In this section we present an empirical data example with a small *N* and a relatively large *p*. As discussed in the previous sections, small *N* and large *p* can cause many problems in estimating and evaluating SEM models. In the illustration we focus specifically on model evaluation with different test statistics, which is a key step for SEM analyses to provide reliable results. The data come from an intervention program for college students who had exhibited depression symptoms. Measurements for both pre- and post-interventions are obtained on *N* = 57 participants, all of whom are college students from universities located in Beijing, China. The data were collected by the first author, as part of a study examining the relationship between resilience and depression.

Resilience was measured by the Connor-Davidson Resilience Scale (CDRISC, Connor and Davidson, 2003). The CD-RISC contains 25 items, with each item rated on a 5-point scale reflecting how a participant felt over the past month, where 1 = Not true at all, 2 = Rarely true, 3 = Sometimes true, 4 = Often true, 5 = True nearly all of the time. The CD-RISC has 3 subscales: toughness (13 items), powerful (8 items), and optimistic (4 items). The model of resilience and depression is formulated with the subscales, not the item scores.

For each participant, measures of depression using the Self-rating Depression Scale (SDS) were also collected. The SDS contains 20 items (Song and Liu, 2013; Zhang et al., 2015; Xu and Li, 2017), with each item rated on a 4-point scale according to how a participant has felt over the past week, where 1 = A little of the time, 2 = Some of the time, 3 = Good part of the time, 4 = Most of the time. Item 2, 5, 6, 11, 12, 14, 16, 17, 18, and 20 were reverse scored items. A higher score on the scale reflects higher levels of depression.

The illustrative data also included six items from “Forgiveness of Others subscale,” which is part of the Heartland Forgiveness Scale (HFS, Thompson et al., 2005). All of the responses are on 7-point Likert scale, with 1–7 signifying responses from “Almost Always False of Me” to “Almost Always True of Me.” Items 1, 3, and 5 were reverse scored items. A higher score indicates more willing to forgive others. While the item-level scores are ordinal variables, for purposes of the illustration, we treat them as continuous variables in the analysis, which leads to very little bias according to Li (2016) and Rhemtulla et al. (2012).

Each participant did the pre-test by filling out the questionnaire before the intervention started, and a post-test 3-months after the group intervention. Thus, we have *p* = 20 variables in total, 10 for the pre-test and 10 for the post-test.

**Figure 1:**
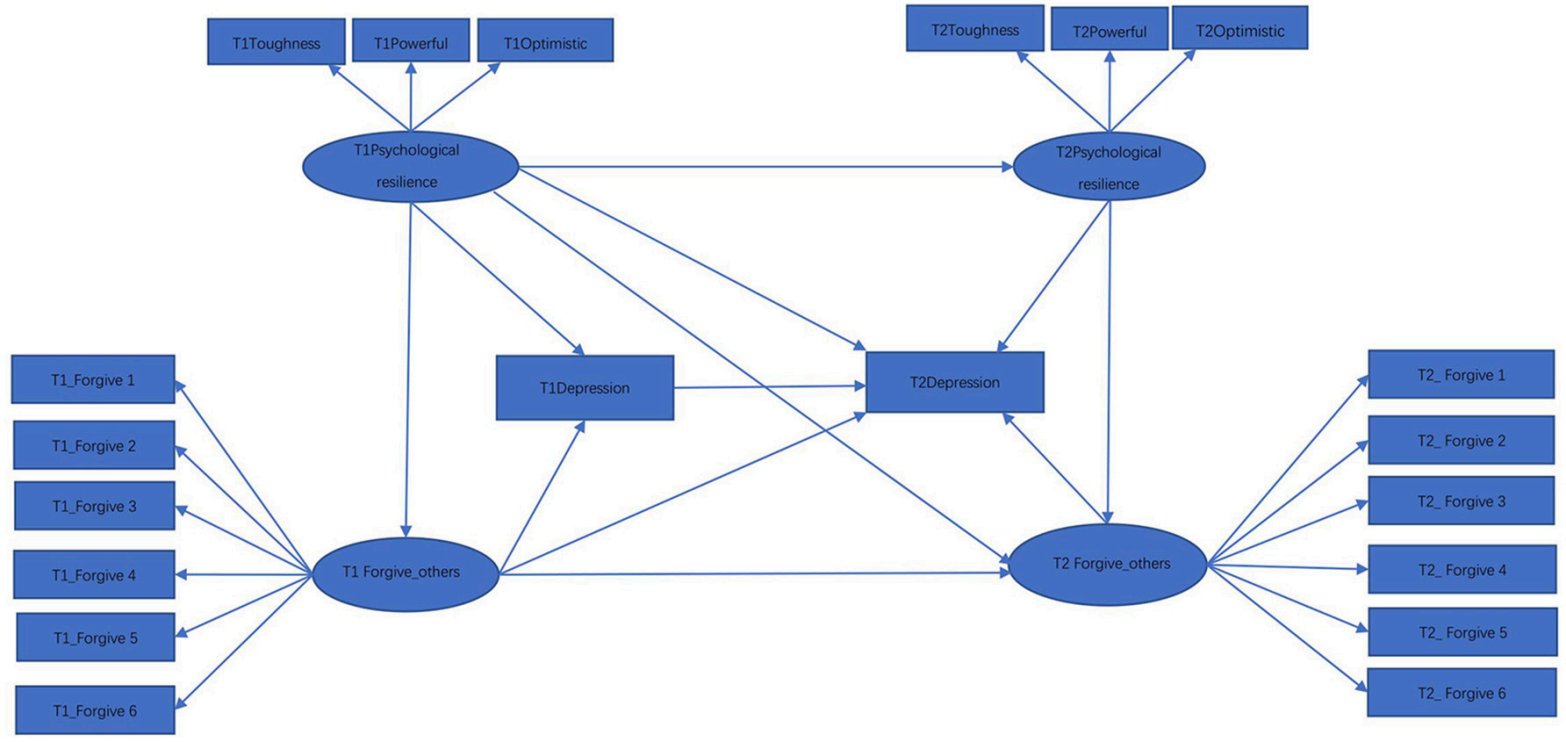
Hypothetical model 1.

Past literature on depression has indicated that a higher level of resilience generally corresponds to a lower level of depression (Kim and Yoo, 2004; Ding et al., 2017; Poole et al., 2017). This literature has also suggested that individuals with depression are expected to be mostly victims of negative events, and that forgiving others is positively correlated with resilience (Dai et al., 2016; Saffarinia et al., 2016). Additionally, it has been determined that a person having a high level of forgiveness is more likely to have a higher level of satisfaction with life, and thus is relatively less depressed (Yu and Zheng, 2008). In accordance with these past research findings, we hypothesize that forgiveness would play a mediating role. Figure 1 is a hypothetical model for exploring the relationship between resilience, depression, and forgiveness of others, where for ease of presentation prediction and measurement errors are not included in the path diagram. We hypothesize that forgiving-others has a mediating effect between psychological resilience and depression. That is, resilience can predict the depression directly, and also can predict depression through forgiving-others. It is also hypothesized that resilience, depression, and forgiveness at time 1 (T1) will have a lasting effect after the group intervention (T2). In addition, the level of resilience at T1 also influences the level of depression and forgiving-others at T2, and the level of forgiving-others at T1 influences the level of depression at T2 as well.

The first line of Table 2 contains the results of the test statistics *T*_*ml*_, *T*_*rml*_ and $T_{rml}^{\left(c20\right)}$ for Model 1, following NML. With *df* = 160, *T*_*ml*_ = 243.97 and *T*_*rml*_ = 242.60 are noticeably statistically significant when compared to $\chi_{160}^{2}$. In contrast, $T_{rml}^{\left(c20\right)}=191.68$, with a corresponding *p* = 0.044, would suggest that Model 1 is close to being adequate.

**Table 2:** Test statistics *T*_*ml*_, *T*_*rml*_ and $T_{rml}^{\left(c20\right)}$ for models 1–4.

**Model**	***df***	***T*_*ml*_**	***p*_*ml*_**	***T*_*rml*_**	***p*_*rml*_**	**${\text{T}}_{\text{rml}}^{\left(\text{c}20\right)}$**	**$p_{\text{rml}}^{\left(\text{c}20\right)}$**
1	160	243.97	2.12 × 10^−5^	242.60	2.72 × 10^−5^	191.68	0.044
2	160	244.29	2.00 × 10^−5^	240.38	4.05 × 10^−5^	190.10	0.052
3	159	225.95	3.84 × 10^−4^	225.33	4.24 × 10^−4^	177.86	0.146
4	159	226.67	3.42 × 10^−4^	224.34	4.96 × 10^−4^	177.20	0.154

**Figure 2:**
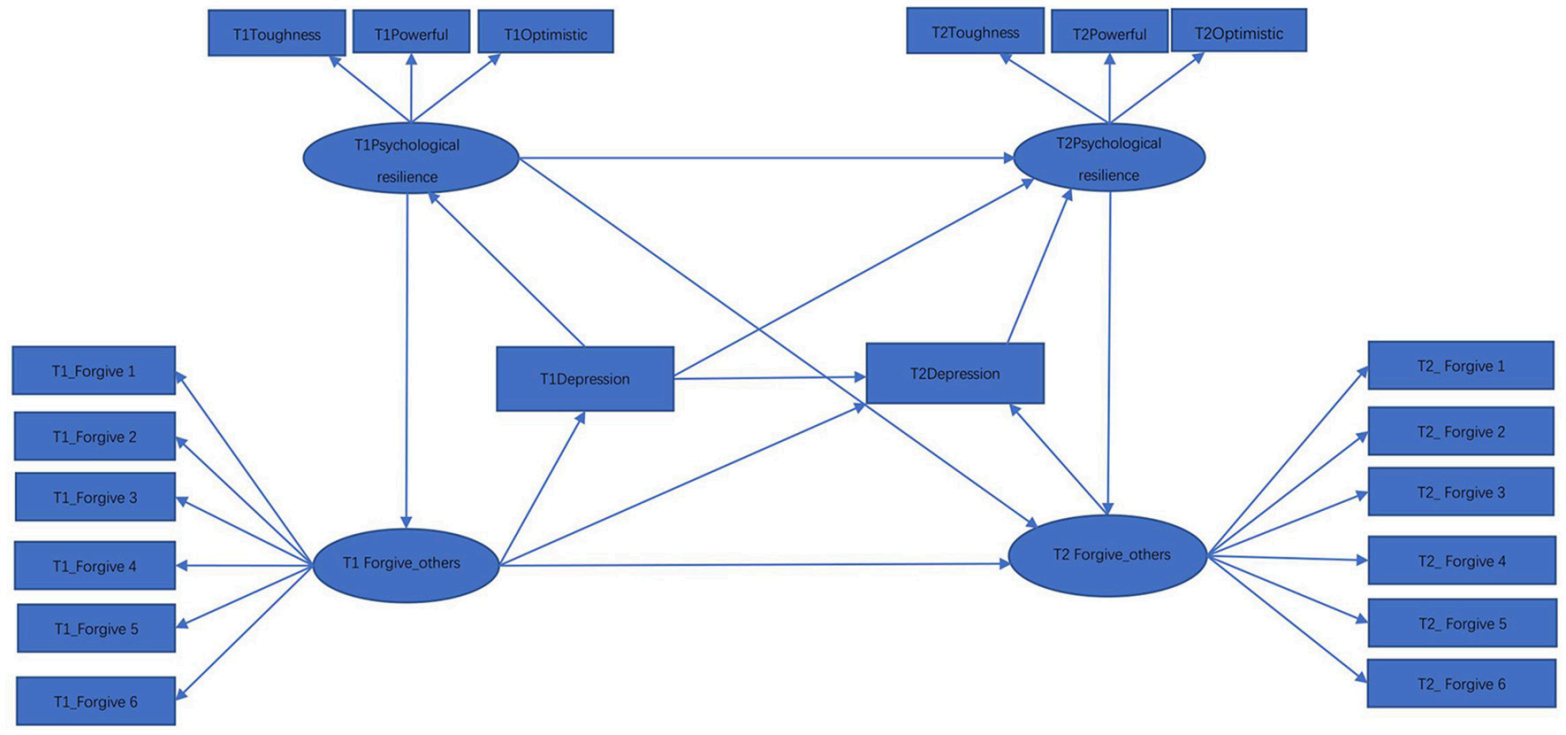
Hypothetical model 2.

While many researchers have showed that psychological resilience can predict depression, numerous studies have also indicated that resilience is influenced by depression (Li et al., 2016; Song et al., 2017; Wang et al., 2017). In particular, these studies have found that resilience of depressed individuals tends to be significantly lower than that exhibited in healthy individuals. Such a hypothetical relationship is represented by Figure 2, where the relationship for the variables between the two time points are set to be identical to those in Figure 1. Test statistics for the model represented by Figure 2 are in the 2nd line of Table 2. While both *T*_*ml*_ = 244.29 and *T*_*rml*_ = 240.38 reject the hypothetical model with *p* < 0.001, $T_{rml}^{\left(c20\right)}=190.10$ suggests that Model 2 is not statistically significant at the nominal level 0.05.

It is important to note that our data are repeated measures, and it is known that such data involving the same measurement across time are likely to have additional correlation due to sharing specific traits. Accordingly, we used a model modification technique to identify the presence of such correlations. Following results obtained by model modification based on the score tests (Sarris et al., 1987), the two errors for indicators Forgive 4 were allowed to correlate in Figure 1, which yielded Model 3. In parallel, allowing the two errors of Forgive 4 in Figure 2 to correlate yielded Model 4. The obtained results for Models 3 and 4 are displayed in the 3rd and 4th lines of Table 2, respectively. For each model, all the three test statistics become less significant. However, the conventional test statistics *T*_*ml*_ and *T*_*rml*_ still reject both models at the level of 0.001. In contrast, the *p*-value corresponding to $T_{rml}^{\left(c20\right)}$ is 0.146 for Model 3 and 0.154 for Model 4, indicating that both models fit the data reasonably well.

In summary, the considered example clearly showed that the most widely used test statistics are no longer reliable for data with large *p* and/or small *N*. By considering the conditions of *N*, *p*, and the empirical distribution, the empirically corrected statistic $T_{rml}^{\left(c20\right)}$ gives the models the appropriate credit they deserve.

## 7. Discussion and conclusions

Data with a small sample size and many variables pose numerous challenges to conventional statistical methods. In this article, we reviewed the developments in SEM for dealing with the issues created by small *N* and/or large *p*. While there are many methods for parameter estimation and overall model evaluation in SEM, only a few can successfully account for the effect of large *p*. Although ridge ML and ridge GLS explicitly accounted for the effect of small *N*, their corresponding test statistics may not follow the nominal chi-square distribution. Among procedures of modeling the sample covariance matrix **S**, the test statistic $T_{rml}^{\left(c20\right)}$ has the mechanism to account for small *N* and the shape of the distribution of the sample. However, it is based on the NML method (which does not have the mechanism to account for small *N*), and may encounter estimation difficulties in practice when the sample covariance matrix is near singular. There is no doubt that additional developments are needed that focus on test statistics following the ridge estimation methods, along with formulas that can yield accurate SEs.

In this article, we mainly reviewed SEM methods based on modeling the sample covariance matrix **S**. When data are non-normally distributed, **S** is not an efficient estimate of **Σ** and the corresponding estimates for the structural parameters are not efficient either. Robust methods for SEM based on robust estimates of **Σ** have been developed (Yuan et al., 2004; Yuan and Zhong, 2008), and these methods are expected to yield more efficient parameter estimates than NML. However, additional developments with the robust methods are needed to deal with the issues of non-convergence and statistics not following the nominal chi-square distributions. Formulas to yield more reliable SEs of the robust estimates also need to be developed. In this article we did not describe methods for dealing with incomplete data, since existing methods for SEM with missing data do not have the mechanism to account for the effect of large *p* and/or small *N* yet (Yuan and Bentler, 2000; Savalei, 2010; Yuan and Zhang, 2012). Additional developments would appear to be needed for SEM with a large number of variables that contain missing data.

In addition to test statistics, fit indices are regularly used for overall model fit evaluation in applications of SEM. Since most popular fit indices are defined via test statistics (e.g., RMSEA, Steiger and Lind, 1980; CFI, Bentler, 1990), they too face the same issues with large *p* and small *N* (e.g., Jackson, 2001). Root mean squared residual (RMSR) is not defined via test statistics, but it needs a proper regulation to be an unbiased estimator of its population counterpart, and the construction of such an unbiased estimator can be especially challenging with small *N* and/or large *p* (Maydeu-Olivares, 2017). Some recent but limited results have shown that the RMSEA and CFI defined via a statistic *T*_*mle*_ does perform much better than their counterparts defined using *T*_*ml*_ (Xing and Yuan, 2017). Based on this work, it is reasonable to expect that advances in test statistics will also improve the performances of other fit indices that are defined via these statistics.

In summary, data with large *p* and small *N* pose a big challenge for SEM methodology and many more new developments are still needed to tackle these issues, especially when the data are incomplete and/or non-normal.

## Author contributions

LD: lead the writing and finalized the article; MY: contributed to computation of the example and the writing of the article; KM: contribute substantially to the writing of the whole article.

### Conflict of interest statement

The authors declare that the research was conducted in the absence of any commercial or financial relationships that could be construed as a potential conflict of interest.

## Footnotes

^1^Factor recovery is commonly measured by a so-called congruence coefficient (https://en.wikipedia.org/wiki/Congruence_coefficient), which is a formula parallel to the product-moment correlation between the population factor loadings and their sample counterparts without centralizing at the mean. Preacher and MacCallum (2002) used root mean squared differences between the population correlations and model implied correlations for measuring factor recovery.
